# Supplementary material for: Repeatable glucocorticoid expression is associated with behavioural syndromes in males but not females in a wild primate
Source: R Soc Open Sci. 2019 Sep 4;6(9):190256. doi: 10.1098/rsos.190256 (PMC6774951; doi:10.1098/rsos.190256)
Supplement: Table S1 [file rsos190256supp1.docx]

**Supplementary Materials**

**Consistent inter-individual differences in glucocorticoid expression are associated with a behavioural syndrome in males but not females in a wild primate**

P. J. Tkaczynski; C. Ross; J. Lehmann; M. Mouna; B. Majolo; A. MacLarnon

**Table S1**: Behavioural variables used to quantify behavioural syndromes in wild Barbary macaques (1). In the first analysis of the data, the repeatability of each variable was examined (results in second column); in the second analysis, factor analysis revealed correlations among the repeatable behavioural variables and these behavioural syndromes were allocated a name based on existing literature and the constituent behaviours (third column). Three repeatable behavioural variables (Activity, Retreats, Body shake) loaded insufficiently on any of the three identified factors. For full definitions of the behavioural variables, refer to (1).

| **Behavioural Variable** | **Repeatable (yes/no)** | **Behavioural Syndrome** |
| --- | --- | --- |
|  |  |  |
| Triadic embrace | Yes | **Excitability** |
| Yawn | Yes |  |
| Embrace | Yes |  |
| Tree shake | Yes |  |
| Open mouth | Yes |  |
| Non-sexual mounting | Yes |  |
| Genital touch | Yes |  |
| Aggression | Yes |  |
| Central | Yes | **Sociability** |
| Neighbors 5-10m | Yes |  |
| Peripheral | Yes |  |
| Self-grooming | Yes | **Tactility** |
| Grooming | Yes |  |
| Neighbors 0-1m | Yes |  |
| Grooming density | Yes |  |
| Activity | Yes | **N/A** |
| Retreats | Yes |  |
| Body-shake | Yes |  |
| Vigilant | No |  |
| Submissions | No |  |
| Supplants | No |  |
| Self-scratch | No |  |
| Teeth chatter | No |  |
| Bare teeth | No |  |
| Lip smack | No |  |
| Approach | No |  |
| Neighbors 1-5m | No |  |
| Grooming diversity | No |  |
|  |  |  |

1. Tkaczynski PJ, Ross C, MacLarnon A, Mouna M, Majolo B, Lehmann J. Measuring personality in the field: An In Situ comparison of personality quantification methods in wild Barbary macaques (Macaca sylvanus). J Comp Psychol. 2018; Advance online publication.
